# PYCR1 inhibition in bone marrow stromal cells enhances bortezomib sensitivity in multiple myeloma cells by altering their metabolism

**DOI:** 10.1002/1878-0261.70120

**Published:** 2025-09-11

**Authors:** Inge Oudaert, Lauren van den Broecke, Osman Aksoy, Judith Lind, Sonia Vallet, Arne Van der Vreken, Gamze Ates, Ann Massie, Ken Maes, Kim De Veirman, Elke De Bruyne, Karin Vanderkerken, Klaus Podar, Eline Menu

**Affiliations:** ^1^ Department of Hematology and Immunology, Myeloma Centre Brussels, Translational Oncology Research Centre Vrije Universiteit Brussel (VUB) Belgium; ^2^ Department General and Translational Oncology and Haematology Karl Landsteiner University of Health Sciences Krems an der Donau Austria; ^3^ Department of Internal Medicine 2 University Hospital Krems Krems an der Donau Austria; ^4^ Centre for Neurosciences, Neuro‐Aging & Viro‐Immunotherapy, Vrije Universiteit Brussel (VUB) Belgium; ^5^ Clinical Sciences, Research Group Genetics, Reproduction and Development, Centre for Medical Genetics Vrije Universiteit Brussel (VUB), Universitair Ziekenhuis Brussel (UZ Brussel) Belgium; ^6^ Department of Clinical Hematology Universitair Ziekenhuis Brussel Belgium

**Keywords:** activin A, multiple myeloma, oxidative phosphorylation, proline metabolism, PYCR1, stromal cells

## Abstract

Despite significant advancements, multiple myeloma (MM) remains incurable, largely due to drug resistance. Our previous research has demonstrated that proline metabolism plays a role in MM progression and that inhibiting PYCR1, the final enzyme in proline synthesis, enhances bortezomib sensitivity in MM cells. Given the high expression of PYCR1 in bone marrow stromal cells (BMSCs), we sought to investigate the effects of PYCR1 inhibition in BMSCs and its indirect influence on MM cell metabolism and viability. Culturing MM cells in conditioned medium (CM) of PYCR1‐silenced BMSC significantly impaired oxidative phosphorylation and sensitised MM cells to bortezomib. Analysis of the CM secretome revealed a reduction in activin A release. Proline and activin A supplementation were able to counteract MM sensitivity to bortezomib. Combination therapy of the PYCR1 inhibitor pargyline and bortezomib reduced tumour load in a 3D model and reduced serum activin A levels in 5TGM1‐bearing mice. This study demonstrates the contribution of stromal cell metabolism to MM progression. Inhibiting PYCR1 in BMSCs leads to less activin A release, limits oxidative phosphorylation in MM cells and enhances bortezomib efficacy.

## Graphical abstract

This study investigated how PYCR1 inhibition in bone marrow stromal cells (BMSCs) indirectly affects multiple myeloma (MM) cell metabolism and viability. Culturing MM cells in conditioned medium from PYCR1‐silenced BMSCs impaired oxidative phosphorylation and increased sensitivity to bortezomib. Secretome analysis of the conditioned medium revealed reduced activin A levels, suggesting a metabolic role for BMSCs in supporting MM cell survival.

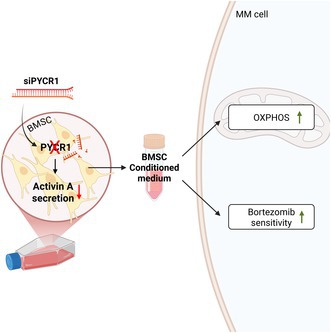

**Abbreviations**
ALL: acute lymphocytic leukaemia
AML: acute myeloid leukaemia
BM: bone marrow
BMPC: bone marrow plasma cells
BMSC: bone marrow stromal cells
Bz: bortezomib
CCLE: cancer cell line encyclopaedia
CLL: chronic lymphocytic leukaemia
CM: conditioned medium
CML: chronic myeloid leukaemia
Combo: combination of pargyline and bortezomib
Ctrl: control
ECAR: extracellular acidification rate
FCCP: carbonyl cyanide‐4‐(trifluoromethoxy)phenylhydrazone
GDH: glutamate dehydrogenase
GLS: glutaminase
GS: glutamate synthetase
hBMSC: human bone marrow stromal cells
MM: multiple myeloma
ND: non detected
Ns: nonsignificant
OAT: ornithine aminotransferase
OCR: oxygen consumption rate
OXPHOS: oxidative phosphorylation
P5CDH: pyrroline‐5‐carboxylate dehydrogenase
P5CS: pyrroline‐5‐carboxylate synthase
PRODH: proline dehydrogenase
PYCR: pyrroline‐5‐carboxylate reductase
Resp.: respiration
Rot/AA: rotenone A/antimycin A
TCA: tricarboxylic acid

## 1 Introduction

Multiple myeloma (MM) is the second most prevalent haematological cancer, surpassed only by lymphoma. MM occurs due to an accumulation of monoclonal plasma cells in the bone marrow [1]. The presence of these abnormal plasma cells and their production of monoclonal antibodies leads to bone lesions, hypercalcemia, anaemia and renal failure [2, 3, 4, 5]. Upon diagnosis, treatment of MM patients consists of a combination therapy of multiple drugs: the proteasome inhibitor bortezomib, the immunomodulating agent lenalidomide, the steroid dexamethasone and the anti‐CD38 monoclonal antibody daratumumab [6, 7]. Although many advances have been made in the last decades, MM remains an incurable cancer due to the occurrence of drug resistance. One of the major factors contributing to this drug resistance is the hypoxic bone marrow microenvironment (TME), which creates a favourable niche for the MM cells.

The TME is a complex network composed of different cell types, extracellular matrix proteins and soluble factors. It actively supports MM cell growth, survival and drug resistance by secreting numerous chemokines, cytokines, growth factors and metabolic substrates. In particular, the bidirectional interaction between MM cells and bone marrow stromal cells (BMSCs) is well studied [8], whereby BMSCs secrete IGF‐1, IL‐6, IL‐13, IL‐1β, VEGF, ß‐FGF, activin A and TNF‐α, to stimulate tumour progression [9, 10, 11].

MM cells alter their metabolism to meet their increasing energy demands [12, 13]. One important metabolic adaptation is the upregulation of the proline biosynthesis, which enhances the conversion of glutamine into proline, a nonessential amino acid essential for protein synthesis and tumour growth [14, 15]. In the mitochondria, glutamine is first converted to glutamate by glutaminase (GLS) and then into pyrroline‐5‐carboxylate (P5C) by pyrroline‐5‐carboxylate synthetase (P5CS). Finally, pyrroline‐5‐carboxylate reductase (PYCR) converts P5C into proline [16, 17]. Three different isoforms of the PYCR enzyme exist: PYCR1 and PYCR2, both localised in the mitochondria, and PYCR3, residing in the cytosol and participating in an alternative pathway for proline production involving ornithine conversion [18, 19, 20].

We found in our previous paper that PYCR1 knockdown in MM cells impaired their proliferation and viability, while potentiating bortezomib‐mediated cell death both *in vitro* and *in vivo* [21]. Moreover, we demonstrated that PYCR1 interference in MM altered PRAS40‐mediated protein synthesis and increased bortezomib sensitivity via the activation of the unfolded protein response pathway [21].

Despite these findings, limited research has been performed on the role of PYCR1 within the TME. Fibroblasts and in particular cancer‐associated fibroblasts (CAFs) are known to synthesise proline to support a tumour‐promoting microenvironment. In breast cancer, Kay *et al*. observed higher levels of PYCR1 in CAFs compared to healthy fibroblasts [22].

In this study, we aimed to further explore the role of PYCR1 in MM pathogenesis by investigating the function of PYCR1 in BMSCs and its role in the metabolic crosstalk with MM cells. To achieve this, we cultured MM cells in conditioned medium (CM) of BMSCs wherein PYCR1 was silenced. The effects of this altered CM on the MM cell viability, apoptosis, metabolic pathway preference and sensitivity to bortezomib were measured. Angiogenesis and cytokine arrays were performed to identify dysregulated chemokines and cytokines in the altered CM. Finally, the effects of inhibiting PYCR1 in BMSCs on tumour progression and its working mechanism were investigated using the small molecule inhibitor pargyline in different coculture setups and an *in vivo* murine experiment.

## 2 Material and methods

### 2.1 Data sets

Gene expression among several cell types in the BM was investigated by consulting the Heidelberg/Montpellier cohort. This cohort contains microarray data of newly diagnosed MM patients as well as healthy volunteers. All data were retrieved through GenomicScape (http://www.genomicscape.com). The MAS5 algorithm was used to normalise the gene expression data. All cell types were isolated from MM patients, except for bone marrow plasma cells (BMPCs), which were derived from healthy controls [23, 24, 25].

### 2.2 Cell culture

The human MM cell line (HMCL) OPM2 (RRID: CVCL_1625) and human stromal cell line HS5 (RRID: CVCL_3720) were obtained from ATCC (Molsheim, France), while the HMCL XG2 (RRID: CVCL_4798) was a kind gift from Jerome Moreaux (Montpellier, France). The murine MM cell line 5TGM1‐eGFP (RRID: CVCL_VI66) was a kind gift from Dr. Kay Oyajobi (Nashville, USA) and the murine stromal cell line MS5 (RRID: CVCL_2128) was a kind gift from Pascale Zimmerman (Leuven, Belgium). We regularly checked the identity of the cell lines by short‐tandem repeat analysis and confirmed mycoplasma negative. HMCLs were cultured in RPMI‐1640 medium (Gibco, Thermo Fisher Scientific, Aalst, Belgium) supplemented with 10% fetal bovine serum (FBS) (Hycone, Logan, UT, USA) and 2 mm l‐glutamine (Gibco). 5TGM1‐eGFP cells were additionally cultured in 1 mm sodium pyruvate (Gibco), 1× MEM NEAA (Gibco), 100 U·mL^−1^ penicillin (Lonza, Basel, Switzerland) and 100 μg·mL^−1^ streptomycin (Lonza), while XG2 cells were additionally cultured in 2 ng·mL^−1^ of recombinant IL‐6 (R&D Systems, Abingdon, UK). HS5 and MS5 cell lines were cultured in DMEM medium supplemented with 10% FBS (Hycone), 2 mm l‐glutamine (Gibco), 1 mm sodium pyruvate (Gibco), 1× MEM NEAA (Gibco), 100 U·mL^−1^ penicillin (Lonza, Basel, Switzerland) and 100 μg·mL^−1^ streptomycin (Lonza). Cell lines were cultured at 37 °C in 5% CO_2_. KM105 human BM stromal cells, a kind gift from Dr. Kenichi (Chiba University Graduate Scholl of Medicine, Chiba, Japan), were cultured in RPMI‐1640 media supplemented with 10% FBS and 100 U·mL^−1^ penicillin (Lonza, Basel, Switzerland) and 100 μg·mL^−1^ streptomycin (Lonza).

### 2.3 Drugs and reagents

Bortezomib (Bz, Selleckchem, Munich, Germany) was dissolved at a stock concentration of 10 mm in dimethylsulfoxide (DMSO, Sigma‐Aldrich, Diegem, Belgium), while pargyline hydrochloride (Sigma‐Aldrich) was dissolved in RPMI‐1640 medium at a stock concentration of 250 mm. L‐proline (Sigma‐Aldrich) was dissolved in Milli‐Q water at a stock concentration of 2 M, while recombinant activin A (R&D Systems, UK) was dissolved at a stock concentration of 100 μg·mL^−1^ in 4 mm of sterile HCl (Sigma‐Aldrich).

### 2.4 Lipofectamine transfection and collection of stromal conditioned medium

HS5 cells were seeded at 300 000 cells per well in a 6‐well plate in DMEM medium, supplemented with 10% FBS, 2 mm l‐glutamine, 1 mm sodium pyruvate, 1× MEM NEAA, 100 U·mL^−1^ penicillin and 100 μg·mL^−1^ streptomycin. One day later, the medium was refreshed to DMEM medium containing 10% FBS and 2 mm l‐glutamine and a transfection mix containing siRNA. SiRNA was purchased from Qiagen (Antwerp, Belgium) and dissolved in sterile RNAse‐free water at a stock concentration of 20 μm. As controls, we used AllStars Negative Control siRNA (1 027 280, Qiagen) (Scrambled) at the same concentration and a mock control. A premix consisting of OptiMEM (Gibco), lipofectamine (Thermo Fisher Scientific, Aalst, Belgium) and siRNA was prepared, mixed and incubated at room temperature for 15 min. Afterwards, the siRNA mixture was added dropwise to the cells. We used a final concentration of 50 nm of siRNA to establish PYCR1 knockdown. The gene‐specific sequences were as follows: PYCR1: sense (5′‐CAC GGG AGC UGC AGU CCA UTT‐3′), antisense (5′‐AUG GAC UGCAGC UCC CGU GTG‐3′). HS5 cells transfected with siRNA were cultured for 72 h, followed by isolation of the CM. After centrifugation, the CM was stored at −20 °C and thawed when needed for experiments.

### 2.5 Collection and experimental use of MM conditioned medium

OPM2 cells were plated at 500 000 cells·mL^−1^ in a T75 flask (75 cm^2^) in RPMI‐1640 medium supplemented with 10% FBS and 2 mm l‐glutamine. After 48 h of incubation, the CM was isolated and centrifuged at 1500 rounds per minute (rpm). After centrifugation, the CM was stored at −20 °C and thawed when needed for experiments.

HS5 cells were seeded one day prior at 300 000 cells per well in a 6‐well plate in DMEM medium supplemented with 10% FBS, 2 mm l‐glutamine, 1 mm sodium pyruvate, 1× MEM NEAA, 100 U·mL^−1^ penicillin and 100 μg·mL^−1^ streptomycin. One day later, the medium was refreshed with fresh RPMI‐1640 medium (supplemented with 10% FBS and 2 mm l‐glutamine), a mixture of RPMI‐1640 medium and OPM2‐CM (= CM 1:2) or OPM2‐CM alone (CM 1). Cell pellets were harvested after 24 h or 48 h.

### 2.6 Viability and apoptosis assays

For viability and apoptotic assays, OPM2 and XG2 cells were seeded at 200 000 cells·mL^−1^ in stromal CM from HS5 (three conditions: mock, scrambled or siPYCR1) in a 24‐well plate and additionally treated with different doses of bortezomib (1 or 1.5 nm). Cells were cultured for 48 h, followed by the CellTiter‐Glo Luminescent Assay to measure cell viability (Promega, Madison, WI, USA), according to the manufacturer's instructions.

To measure apoptotic cell death, we harvested the cells, washed them with FACS flow and stained them with a mixture of 98 μL of 1× binding buffer, 1 μL of Annexin V‐APC staining and 1 μL of 7‐AAD staining per sample (all from BD Biosciences, Erembodegem, Belgium). After 15 min of incubation in the dark, 300 μL of 1× binding buffer was added. Samples were analysed by flow cytometry on a BD Accuri C6 flow cytometer (BD Biosciences).

For coculture experiments, MS5 cells were seeded at 50 000 cells/well in a 24‐well plate in DMEM medium supplemented with 2 mm l‐glutamine. For immediate combination treatment of pargyline and bortezomib, 100 000 5TGM1‐eGFP cells were added per well and treated with 2 mm of pargyline and/or 2.5 nm of bortezomib. Viability, as indicated by eGFP intensity, was measured by flow cytometry on a BD Accuri C6 flow cytometer after 48 h of incubation. For pretreatment experiments, MS5 cells were treated with 2 mm of pargyline. After 48 h, 100 000 5TGM1‐eGFP cells were added per well and treated with 3 nm of bortezomib. 48 h later, viability (as indicated by the eGFP positivity) was measured by flow cytometry on a BD Accuri C6 flow cytometer.

### 2.7 Seahorse metabolic analysis

XFe96‐well plates (Agilent Technologies, Zaventem, Belgium) were coated with 0.01% poly‐L‐lysine (molecular weight 150 000‐300 000; Sigma‐Aldrich) one day prior to the assay and incubated overnight at 37 °C. One day later, the coated plate was washed three times with sterile Milli‐Q water and used for the experiment.

OPM2 cells were seeded at 200 000 cells·mL^−1^ in a 6‐well plate and treated with HS5‐CM (mock, scrambled or siPYCR1) and/or 1 nm bortezomib. After 24 h (combinations with bortezomib) or 96 h (CM mock, scrambled or siPYCR1) of incubation, cells were isolated, centrifuged and washed twice in Seahorse DMEM assay medium (supplemented with 2 mm glutamine, 1 mm pyruvate and 10 mm glucose) and counted. Cells were seeded at 50 000 cells/well in Seahorse DMEM medium in the precoated plate. The Seahorse Mito Stress test assay (Agilent) was performed according to the manufacturer's instructions. We used the following final inhibitor concentrations in each well: 1.5 μm oligomycin, 1 μm carbonyl cyanide‐4‐phenylhydrazone (FCCP), 0.5 μm antimycin A and 0.5 μm rotenone. After completion of the assay and real‐time measurements of oxygen consumption rates (OCR) and extracellular acidification rates (ECAR), we stained the cells with Hoechst for normalisation. Automatic cell counting was performed using the cytation1 cell imaging multimode reader (BioTek instruments). Data were normalised to cell number.

### 2.8 CD138+ and CD138‐ cell isolation from human bone marrow samples

BM samples of MM patients were collected for routine diagnostic or evaluation purposes after their written informed consent and in accordance with the Declaration of Helsinki and institutional research board approval from Brussels University Hospital (B.U.N. 143 201 838 414). A density gradient centrifugation procedure (Lymphoprep™, Catalog # 07801, STEMCELL™ technologies, Grenoble, France) was used to isolate the mononuclear cells. To separate the CD138+ cells (= MM cells) from the CD138‐ cells, cells were labelled with human CD138 Microbeads (order no. 130–051‐301, Miltenyi Biotec, Gladbach, Germany) and isolated by magnetically activated cell sorting according to the manufacturer's instructions.

### 2.9 Culture of human primary bone marrow stromal cells

BM samples of MM patients were collected and processed as described above. BM samples of MM patients were collected for routine diagnostic or evaluation purposes after their written informed consent and in accordance with the Declaration of Helsinki and institutional research board approval from Brussels University Hospital (B.U.N. 143 201 838 414). CD138‐ cells were cultured in DMEM medium, supplemented with 10% FBS, 10% horse serum (Gibco), 2 mm l‐glutamine, 1 mm sodium pyruvate, 1× MEM NEAA, 100 U·mL^−1^ penicillin and 100 μg·mL^−1^ streptomycin. Cells were initially cultured for 48h‐72h and then refreshed with fresh medium to remove all nonattaching cells. Cells that started proliferating after the first refreshment were considered primary BMSCs. Cells were not passaged more than 6 times when used in experiments.

### 2.10 Western blot

OPM2 cells were seeded into a 6‐well plate, treated with CM of BMSCs and cultured for 48 h (combination with bortezomib) or 72 h (no combination with bortezomib). Cells were collected, centrifuged at 1500 rpm for 5 min, and the supernatant was removed. The cell pellet was lysed in a lysis buffer, which consists of 50 mm Tris, 150 mm NaCl, 1% Nonidet P40, and 0.25% sodium deoxycholate, supplemented with the following protease and phosphatase inhibitors: 4 mm Na_3_VO_4_, 1 mm Na_4_P_2_O_7_, 2 μg·mL^−1^ aprotinin, 50 μg·mL^−1^ leupeptin, 500 μg·mL^−1^ trypsin inhibitor, 10 μm benzamidine, 2.5 mm pnp benzoate (all from Sigma‐Aldrich), 50 mm NaF, 5 mm ethylenediaminetetraacetic acid (both from VWR International, Radnor, PA, USA), 1 mm 4‐(2‐aminoethyl) benzenesulfonyl fluoride hydrochloride, and 50 μg·mL^−1^ pepstatin A (both from ICN, Costa Mesa, CA, USA). Pellets were kept on ice for 10 min, followed by high‐speed centrifugation at 14000 rpm. The supernatant was collected and stored at −80 °C. Protein concentration was determined using bicinchoninic acid (BCA) assay. Western blotting was performed as previously described [26]. Chemiluminescence was visualised (Plus‐ECL, PerkinElmer, Mechelen, Belgium) and determined using Li‐Cor Odyssey Fc (Bad Homburg, Germany). Antibodies used for analysis were as follows: β‐ACTIN (#4967), p‐AKT (#4056, phosphorylation site Thr308), AKT (#9272), caspase 3 (#9665), PARP (#9542, all from Cell Signalling Technologies, Leiden, The Netherlands) and PYCR1 (13108‐1‐AP, Proteintech, Manchester, UK). We used a horseradish peroxidase (HRP)‐coupled anti‐rabbit antibody (#7074, Cell Signalling Technology, dilution 1:5000) as a secondary antibody.

### 2.11 Quantitative real‐time PCR

Cells were isolated and centrifuged at 1500 rpm for 5 min, followed by supernatant removal. We extracted and purified total RNA using the NucleoSpin RNA plus kit (Macherey‐Nagel, Düren, Germany). About 1 μg of RNA was converted to cDNA by the Verso cDNA Synthesis Kit (Thermo Fisher Scientific, Waltham, MA, USA) in a final volume of 20 μL, consisting of 11 μL of RNA sample (1000 ng·μL^−1^), 4 μL of 5× cDNA synthesis buffer, 2 μL of deoxynucleoside triphosphate (dNTP) mix, 1 μL of RNA primer, 1 μL of RT enhancer and 1 μL of Verso enzyme mix. Next, the mixture was incubated for 30 min at 42 °C, followed by 2 min at 95 °C in a SWIFT™ MiniPro Thermal Cycler (Esco). Afterwards, we performed real‐time PCR using SYBR Green (PowerUp™ SYBR™ Green Master Mix, Applied Biosystems, Thermo Fisher Scientific, Dilbeek, Belgium) in a final volume of 25 μL, consisting of 1 μL of cDNA, 12.5 μL of SYBR Green, 1 μL of Primer Mix 10 pmol·μL^−1^ and 10.5 μL of nuclease‐free water. Gene‐specific primer sequences were as follows: pycr1: forward (5′‐GTG GTT ACT GTG GGT GGA ATA‐3′), reverse (5′‐CAG ATG CCC TCC AAG ATG TG‐3′), ß‐actin: forward (5′‐CAC TCT TCC AGC CTT CCT TC‐3′), reverse (5′‐GTA CAG GTC TTT GCG GAT GT‐3′), activin a: forward (5′‐TCT GCA GTA GTG TGG ACT AGA A‐3′), reverse (5′‐CCT GGG TAA TTG GGT AGG AAA G‐3′), il13: forward (5′‐CAC GGT CAT TGC TCT CAC TT‐3′), reverse (5′‐CTT CTG GTT GAT GTT‐3′), il1b: forward (5′‐CAA AGG CGG CCA GGA TAT AA‐3′), reverse (5′‐CTA GGG ATT CAG TCC ACA TTC AG‐3′). All primers were purchased from Integrated DNA Technologies (Leuven, Belgium). We used the QuantStudio 5 Flex Real‐Time PCR System (Thermo Fisher Scientific) to quantify the expression level of mRNA. The housekeeping gene ß‐actin was used for data normalisation. The comparative ΔΔCt method was used to investigate differential expression compared to a control sample.

### 2.12 Proteome profiling of the stromal conditioned medium

CM of HS5 cells with or without PYCR1 silencing was isolated after 72 h of culture. We identified relative levels of selected angiogenesis‐related proteins, cytokines and chemokines using the Human Cytokine (ARY005B) and Human Angiogenesis Array kit (ARY007, both from R&D Systems) according to the manufacturer's instructions. We visualised and analysed the levels of the different proteins, cytokines and chemokines using the Li‐COR Odyssey Fc. Image Studio Lite version 5.2 software was used for quantification.

### 2.13 3D scaffold coculture setup and confocal microscopy

The 3D scaffold coculture system was generated as previously described [27]. Shortly, KM105 (RRID: CVCL_DE28) stromal cells were stained with Qtracker^®^ 625 (Thermo Fisher Scientific) as per manufacturer instructions. Poly‐Ɛ‐caprolactone (PCL) scaffolds (3D BioTek^®^, Bridgewater, NJ, USA) were each seeded with 100 000 labelled KM105 cells in a 96‐well plate and incubated overnight at 37 °C. The next day, 3 000 000 OPM2‐eGFP+ cells were added directly onto the scaffold in the 96‐well plate and incubated at 37 °C for 4 h. Afterwards, scaffolds were carefully transferred to the vessels of the Rotary Cell Culture System™ (Synthecon Inc. Houston, TX, USA) and treated with 10 nm bortezomib and/or 2 mm pargyline in RPMI‐1640 medium supplemented with 2% FBS and 2 mm l‐glutamine as described.

After 48 h, scaffolds were isolated in 96‐well plates, washed with PBS and fixed in 3% paraformaldehyde for 30 min at room temperature. Next, the 96‐well plate was kept at 4 °C until staining and microscopy. The supernatant in the vessels was also isolated, centrifuged at 1500 rpm and counted, followed by fixation and storage as described above. For confocal microscopy, scaffolds were additionally stained with DAPI and collagen VI‐AF647 (Abcam) and imaged using confocal microscopy Leica TCS SP8 X (Vienna, Austria). We visualised and analysed the images using imagej [28].

### 2.14 Serum collagen C‐terminal telopeptide quantification

A murine experiment in C57BL/KalwRij mice was performed using 5TGM1‐eGFP+ MM cells to evaluate the clinical potential of the PYCR1 inhibitor pargyline. The experiment was performed as previously described [21]. Briefly, mice were treated with either bortezomib, pargyline or a combination of both agents. After 30 days, vehicle mice reached end‐stage and all mice were sacrificed. The housing and treatment of the mice were approved by the Ethical Committee for Animal Experiments of the Vrije Universiteit Brussel (licence No LA1230281, CEP No 20‐281‐6). We isolated blood from the retro‐orbital plexuses, allowed the blood to clot for 30 min, followed by high‐speed centrifugation at 14 000 rpm for 5 min. Afterwards, serum was collected and stored at −20 °C. We performed an ELISA to measure circulating collagen type I degradation product in the serum of the mice, using the Mouse CTX/Collagen C‐terminal telopeptide ELISA kit (LifeSpan BioSciences, Inc., Seattle, WA, USA) according to manufacturer's instructions. Before the assay, serum samples were diluted 1:10.

### 2.15 Serum activin A quantification

We performed an ELISA to measure activin A levels in the serum of mice. The serum was isolated and stored as mentioned above. The assay was performed according to the manufacturer's instructions (#DAC00B, R&D Biosystems).

### 2.16 Statistical analysis

We analysed the results using GraphPad Prism 10.0 software (GraphPad Software Inc, La Jolla, CA, USA). All data are shown as the mean value ± standard deviation. We performed a Mann–Whitney U test or a one‐way ANOVA test. *P* ≤ 0.05 (*), *P* ≤ 0.01 (**), *P* ≤ 0.001 (***) and *P* ≤ 0.0001 (****) were considered statistically significant.

## 3 Results

### 3.1 PYCR1 is highly expressed in MM cells and BMSCs

**Fig. 1:**
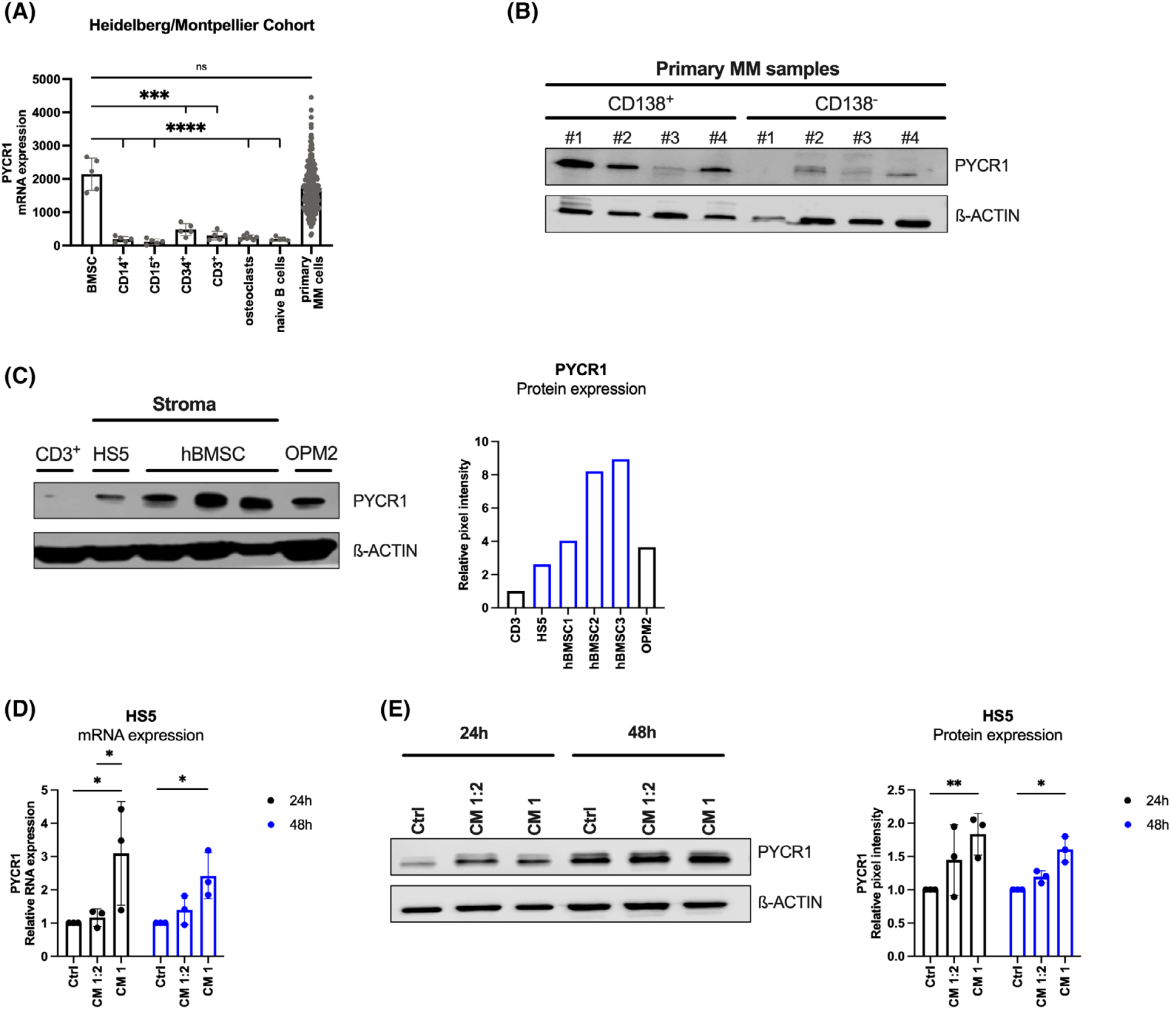
PYCR1 is highly expressed in MM cells and bone marrow stromal cells (BMSCs). (A) PYCR1 gene expression was investigated by consulting the Heidelberg–Montpellier cohort, consisting of microarray data of newly diagnosed MM patients and healthy volunteers. (BMSC, CD14^+^, CD15^+^, CD34^+^, CD3^+^, naïve B cells: *n* = 5; osteoclasts: *n* = 7; primary MM cells: *n* = 206). (B) Western blot analysis of PYCR1 protein expression in matched CD138+ and CD138‐ fractions of freshly isolated primary MM bone marrow samples (*n* = 4). (C) Western blot analysis of PYCR1 protein expression in BMSC cell line HS5 and cultured primary stromal cells from three different MM patients compared to the levels of CD3^+^ T cells (low PYCR1 expression levels) and OPM2 cells (high expression levels) (*n* = 1). (D) PYCR1 mRNA expression as measured by RT‐qPCR of HS5 cells treated with OPM2 conditioned medium (CM) for 24 h and 48 h (*n* = 3). (E) Western blot analysis of PYCR1 expression of HS5 cells treated with OPM2‐CM for 24 h and 48 h (*n* = 3). Statistical significance was determined by a one‐way ANOVA. *P* < 0.05 (*), *P* < 0.01 (**), *P* < 0.001 (***), *P* < 0.0001 (****). Data are presented as mean ± SD. CM, conditioned medium; Ctrl, control; hBMSC, human bone marrow stromal cells; MM, multiple myeloma; PYCR1, pyrroline‐5‐carboxylate reductase 1.

We first evaluated PYCR1 expression in the different cell types present in the tumour microenvironment of MM patients consulting the Heidelberg/Montpellier cohort. High PYCR1 expression was detected in primary MM cells and surprisingly also in bone marrow stromal cells (BMSCs). In contrast, lower expression of PYCR1 was found in CD14+ monocytic cells, CD15+ granulocytic cells, CD34+ hematopoietic cells, CD3+ T cells, osteoclasts and naïve B cells (Fig. 1A).

To confirm the high expression of PYCR1 on protein levels in primary samples, BM samples of different MM patients (*n* = 4) were collected and separated into CD138+ (= MM cells) and CD138‐ fractions (= all other cell types) fractions. High protein expression of PYCR1 was observed in MM cells (CD138+), while lower expression was present in CD138‐ cells (Fig. 1B). To validate PYCR1 expression in primary BMSCs, several CD138‐ fractions were cultured *in vitro* and passaged over time. Only the adherent cells were able to proliferate and were considered primary BMSCs. When comparing PYCR1 expression across different cell types, strong PYCR1 expression was observed in both primary BMSCs and the human BM stromal cell line HS5, relative to the high expression in OPM2 cells and low expression in CD3+ T cells (Fig. 1C).

Finally, we evaluated whether the secretome of MM cells could modulate PYCR1 expression in BMSCs. We treated HS5 cells (BMSC cell line) with the conditioned medium (CM) of OPM2 cells (MM cell line) and assessed the expression levels of PYCR1 in HS5 stromal cells on both RNA (Fig. 1D) and protein level (Fig. 1E). HS5 cells were treated with two different concentrations of CM: either a mixture of 50% CM and 50% fresh medium (CM 1:2) or complete CM (CM 1). Treatment with complete CM significantly increased PYCR1 expression on both RNA and protein levels after 24 h and 48 h. While CM 1:2 did not upregulate PYCR1 at the RNA level, we did observe a small but significant increase in PYCR1 protein expression in stromal cells after 48 h of culture. These findings highlight that MM cell‐derived secretome can upregulate PYCR1 expression in BMSCs, which suggest a potential role for PYCR1 in modulating the pro‐tumorigenic microenvironment.

### 3.2 PYCR1 inhibition in BMSCs modulates mitochondrial respiration in MM cells

Given that PYCR1 expression is elevated in BMSCs and further increases in response to stimulation by the MM cell secretome, we investigated whether stromal PYCR1 expression also contributes to tumour progression and drug resistance. HS5 cells were transfected with siRNA against PYCR1 (siPYCR1) or controls (mock and scrambled) and PYCR1 knockdown was confirmed at 48 h on both RNA and protein levels (Fig. S1A,B). No effect on cell viability was observed at this timepoint in HS5 cells upon PYCR1 silencing (Fig. S1C). PYCR1 knockdown was shown to be stable up to 96 h at the RNA level (Fig. S1D).

**Fig. 2:**
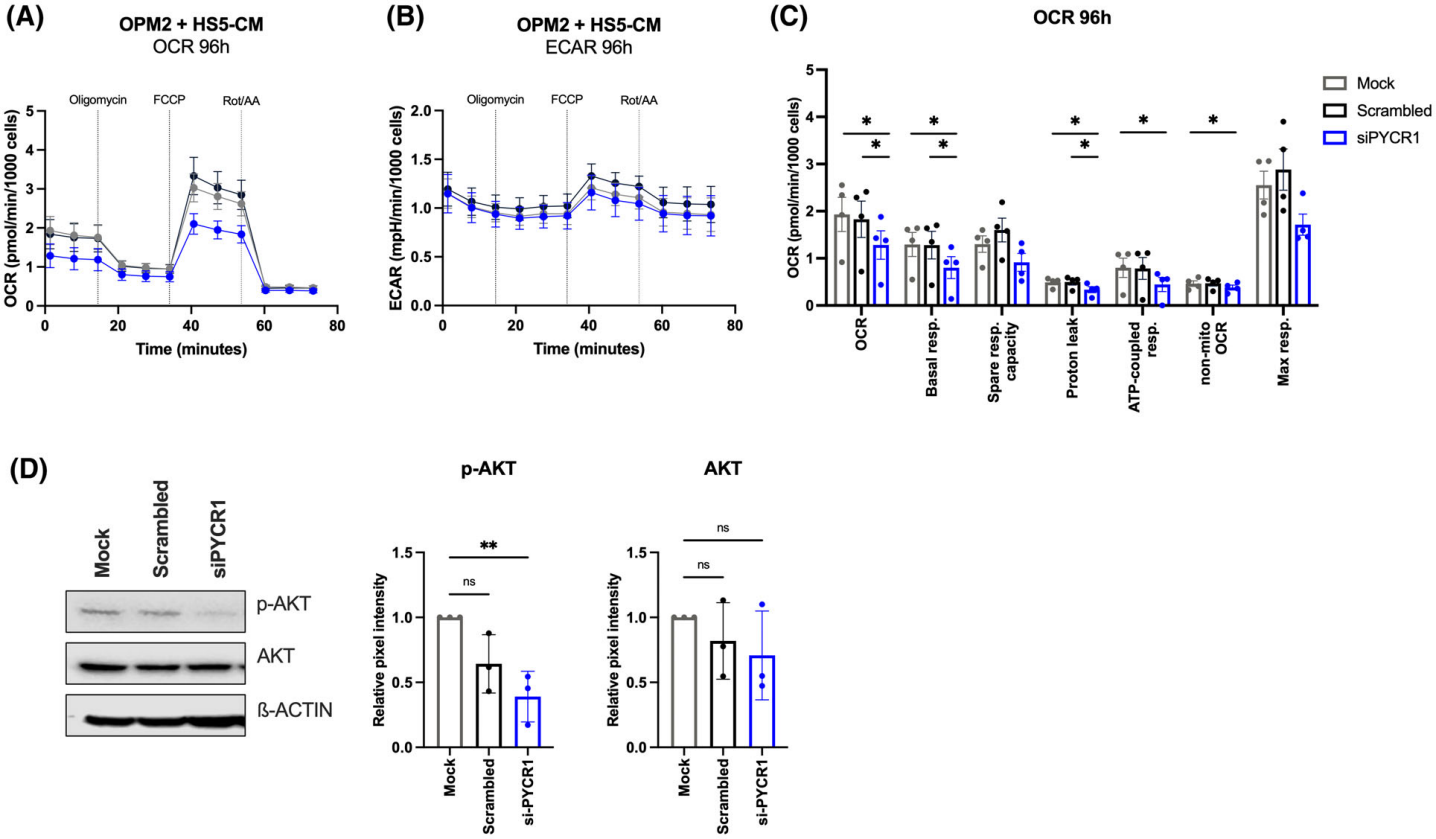
PYCR1 inhibition in BMSCs reduces OXPHOS, but not glycolysis, in MM cells as indicated by reduced OCR. (A, B) OCR and ECAR rates measured in real time after OPM2 cells were treated for 96 h with CM isolated from PYCR1‐inhibited HS5 cells (siPYCR1‐CM) or control (mock, scrambled) (*n* = 4) using the Seahorse Mito Stress Test Technology. (C) Overview of multiple OXPHOS‐related parameters (*n* = 4). (D) Western blot analysis of AKT proteins in OPM2 cells measured after 72‐h treatment with CM isolated from PYCR1‐inhibited stromal HS5 cells (siPYCR1) or control (mock, scrambled) (*n* = 3). Statistical analysis was performed using a one‐way ANOVA test. *P* < 0.05 (*), *P* < 0.01 (**). Data are presented as mean ± SD. OXPHOS, oxidative phosphorylation; CM, conditioned medium; ns, nonsignificant; OCR, oxygen consumption rate; ECAR, extracellular acidification rate; Rot/AA, otenone/antimycin A; FCCP, phenylhydrazone; non‐mito, nonmitochondrial; resp., respiration.

As PYCR1 is a key enzyme in proline biosynthesis, we investigated whether CM of PYCR1‐inhibited BMSCs cells (siPYCR1‐CM) would induce metabolic alterations in the MM cells. Therefore, we cultured MM cells in control CM (mock‐CM or scrambled‐CM) or siPYCR1‐CM, after which we measured oxygen consumption rate (OCR) as an indicator for oxidative phosphorylation (OXPHOS) using the Seahorse Mito Stress test technology. After 96 h of treatment with siPYCR1‐CM, OPM2 cells exhibited lower OCR rates compared to their control CMs (Fig. 2A). In contrast, the extracellular acidification rates (ECAR), an indicator of glycolysis, remained unchanged, suggesting that glycolytic activity was not influenced by PYCR1 inhibition (Fig. 2B). More specific analysis of the OCR changes revealed significant decreases in OCR, basal respiration, proton leak, ATP‐linked respiration and nonmitochondrial OCR in OPM2 cells upon treatment with siPYCR1‐CM (Fig. 2C). The PI3K pathway has been found to regulate OXPHOS [29]. When examining this pathway, we found that phosphorylation of Akt (p‐AKT) was significantly inhibited when OPM2 cells were cultured in siPYCR1‐CM (Fig. 2D).

### 3.3 PYCR1 silencing in BMSCs enhances bortezomib‐mediated effects in MM cells in coculture

**Fig. 3:**
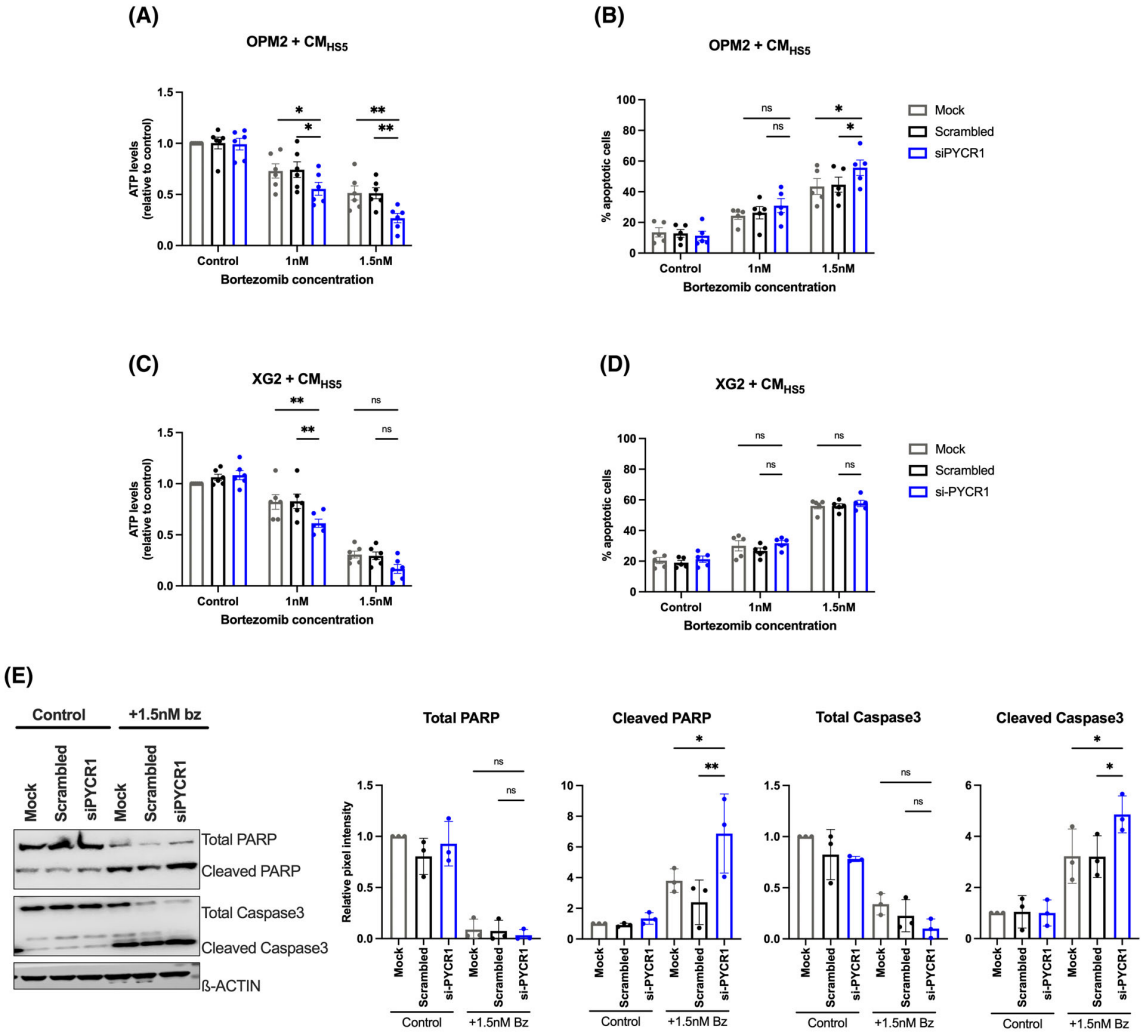
PYCR1 inhibition in BMSCs sensitises MM cells to bortezomib treatment in coculture. OPM2 and XG2 cells were cultured in the presence of HS5‐CM, with or without PYCR1 knockdown (mock, scrambled, siPYCR1) for 48 h and simultaneously treated with 2 different bortezomib concentrations. (A) Viability in OPM2 was measured by CellTiter‐Glo assay (*n* = 6). (B) Apoptotic cell death in OPM2 was quantified by Annexin V and 7‐AAD staining on flow cytometry (*n* = 5). (C)Viability in XG2 was measured by CellTiter‐Glo assay (*n* = 6). (D) Apoptotic cell death in XG2 was quantified by Annexin V and 7‐AAD staining on flow cytometry (*n* = 5). (E) Cell pellets were isolated, proteins extracted and western blot was performed and quantified for PARP and caspase 3 expression levels (*n* = 3). Statistical analysis was performed using a one‐way ANOVA test. *P* < 0.05 (*), *P* < 0.01 (**). Data are presented as mean ± SD. bz, bortezomib; ns, nonsignificant.

BMSCs are known to protect MM cells against bortezomib and it has been shown that bortezomib‐resistant MM cells exhibit increased mitochondrial respiration [30, 31, 32] and increased Akt activation [33]. Therefore, we investigated whether reduced Akt‐OXPHOS activation in MM cells when treated with siPYCR1‐CM could affect their sensitivity to bortezomib. We treated MM cells with 2 different concentrations (1 nm or 1.5 nm) of bortezomib in combination with stromal PYCR1 silencing. This combination significantly decreased the viability of both OPM2 and XG2 cells compared to bortezomib monotherapy (Fig. 3A,C). Additionally, we silenced PYCR1 in HT1080, a fibrosarcoma cell line, and observed no impact on the bortezomib sensitivity in MM cells. This shows that the effects are specific to the MM‐BMSC interaction and are not mediated by other cell types within the TME (Fig. S2B,C). The combination treatment significantly increased apoptotic cell death in OPM2 and up to 10% in XG2 (Fig. 3B,D). This was further supported by a significant increase in cleaved PARP/total PARP and cleaved caspase 3/total caspase 3 levels (OPM2, Fig. 3E).

Given that stromal siPYCR1‐CM reduced OCR in MM cells and that the combination therapy with bortezomib further lowered viability, we examined whether the combination therapy also further affected OCR. A reduction in OCR values for OPM2 cells treated with a combination of siPYCR1‐CM and bortezomib was observed at 24 h, following carbonyl cyanide‐p‐trifluoromethoxyphenylhydrazone (FCCP) injection (Fig. S3A). Additionally, bortezomib treatment increased ECAR levels, indicative of elevated glycolysis; however, this effect was reversed in MM cells when they were treated with bortezomib and siPYCR1‐CM (Fig. S3B). No effects on other mitochondrial respiration parameters were observed due to high variations (Fig. S3C). These findings suggest that stromal PYCR inhibition, when combined with bortezomib, not only enhanced apoptosis at 48 h, but that this could be by prior impairment of mitochondrial function, seen at 24 h.

### 3.4 Silencing of PYCR1 in BMSCs reduces activin A and proline release into its conditioned medium

**Fig. 4:**
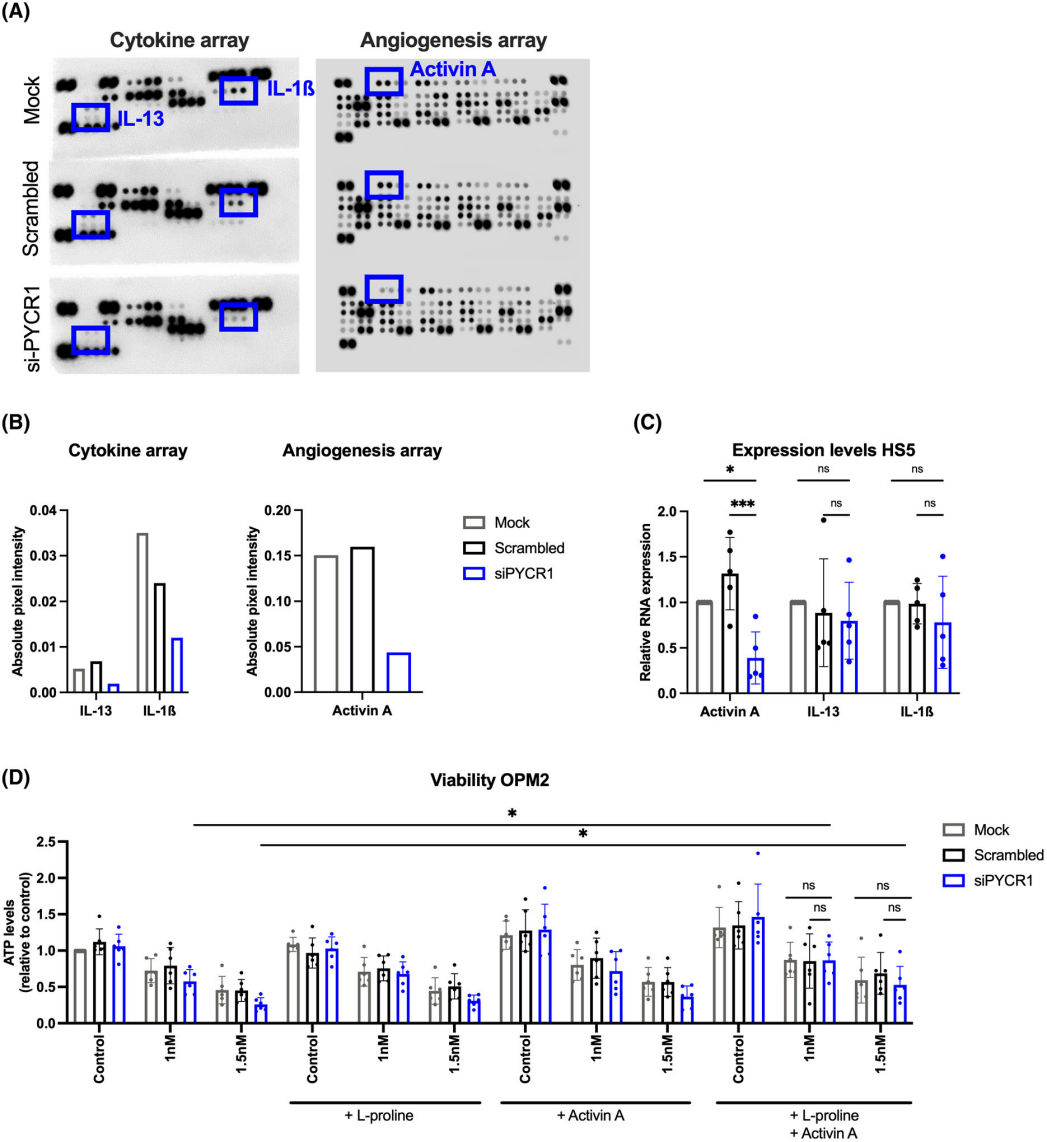
PYCR1 silencing in BMSCs reduces activin A release into the medium. (A‐B) Cytokine and angiogenesis array and analysis of CM isolated from HS5 cells with (siPYCR1) or without PYCR1 silencing (mock, scrambled). Chemiluminescence was measured using Li‐Cor Odyssey Fc, and quantification was performed using Image Studio Lite version 5.2 software (*n* = 1). (C) RNA expression of activin A, IL‐13 and IL‐1ß in HS5 cells with or without PYCR1 silencing as measured by RT‐qPCR (*n* = 5). (D) Viability of OPM2 cells treated with CM from PYCR1‐inhibited HS5 cells (siPYCR1) or control (mock, scrambled), along with bortezomib (1 or 1.5 nm), proline (1 mm), activin A (100 μg·mL^−1^) or a combination of proline and activin A. Viability was measured by CellTiter‐Glo^®^ after 48 h of incubation (*n* = 6). Statistical analysis was performed using a one‐way ANOVA test. *P* < 0.05 (*), *P* < 0.01 (**), *P* < 0.001 (***). Data are presented as mean ± SD. CM, conditioned medium; ns, nonsignificant.

To assess whether the reduction in bortezomib sensitivity observed with siPYCR1‐CM was mediated through a change in the BMSCs secretome, we performed a cytokine and angiogenesis array to identify any possible dysregulated factors. The CM of mock‐, scrambled‐ and siPYCR1‐treated BMSCs cells were tested, and we observed a decrease in activin A, IL‐13 and IL‐1ß in the siPYCR1‐CM (Fig. 4A,B). We also confirmed the decrease in activin A on mRNA levels in HS5 stromal cells, while the decrease was not found significant for IL‐13 and IL‐1ß due to high standard deviations (Fig. 4C).

We then performed rescue experiments by re‐supplementing the siPYCR1‐CM with activin A and proline. We treated the OPM2 cells with the different HS5 CMs (mock, scrambled, siPYCR1), alongside bortezomib, proline and activin A. A reduction in cell viability (17% at 1 nm and 43% at 1.5 nm) was observed when bortezomib was added in the presence of siPYCR1‐CM, and this reduction was restored (90–100% rescue) to the levels of Mock‐CM when both proline and activin A were re‐supplemented (Fig. 4D). We found that the combination of proline and activin A significantly increased (1.48‐fold and 2‐fold) viability of the bortezomib‐treated cells, compared to 1 nm and 1.5 nm monotherapy, respectively. These findings suggest that the depletion of proline and activin A in the siPYCR1‐CM contributes to the observed decrease in cell viability and that these factors play a role in modulating MM cell survival.

### 3.5 PYCR1 inhibition enhances bortezomib sensitivity *in vitro* and reduces activin A release *in vivo*

We continued by investigating the therapeutic potential of targeting PYCR1 in the tumour microenvironment in a clinical setup. We evaluated the combination strategy using the PYCR1 inhibitor pargyline with bortezomib in a relevant 3D scaffold system. In this model, BMSCs were seeded directly onto the scaffold, followed by the addition of OPM2‐eGFP+ cells 24 h later. Afterwards, the scaffolds were transferred to the vessels of a rotating system and incubated with medium, with either the PYCR1 inhibitor pargyline or bortezomib or a combination of both for 48 h. The scaffolds and CM were isolated for further analysis using confocal microscopy.

**Fig. 5:**
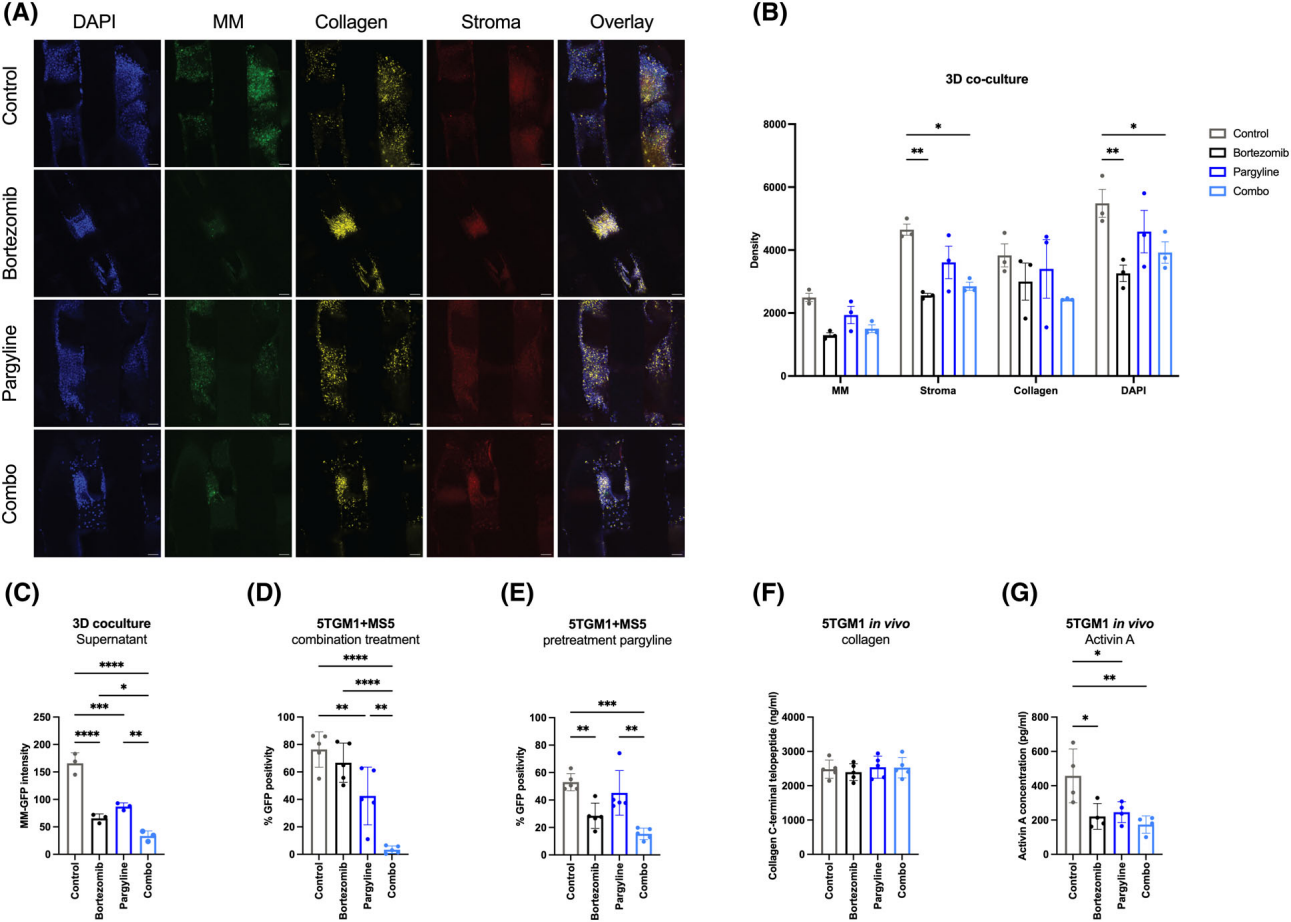
PYCR1 inhibition by pargyline enhances bortezomib sensitivity in vitro and reduces activin A release *in vivo*. (A, B) 3D scaffold coculture of OPM2 MM cells and KM105 stromal cells, treated with bortezomib, pargyline or a combination of both (one representative of 3 is shown). Densities were measured after 48 h of continuous rotation on the Rotary Cell Culture system and analysed by confocal microscopy. Scale bar is represented as a line on each figure (Scale bars = 100 μm). (C) OPM2‐GFP cell density in the supernatant of the 3D coculture system. (D) GFP intensity was measured in a coculture setting of MS5 and 5TGM1‐eGFP‐positive cells after 48 h of treatment with 3 nm bortezomib and/or 2 mm pargyline (*n* = 5). (E) GFP intensity was measured in a coculture setting of MS5 and 5TGM1‐eGFP‐positive cells after 48 h of MS5 pretreatment with 2 mm pargyline, followed by coculture with 5TGM1 cells and 3 nm bortezomib treatment (*n* = 5). (F) Collagen C‐terminal telopeptide concentrations in serum of 5TGM1‐bearing mice treated with bortezomib, pargyline or a combination of both, measured by ELISA (*n* = 5). (G) Activin A concentrations in serum of 5TGM1‐bearing mice treated with bortezomib, pargyline or both, measured by ELISA (*n* = 3). Statistical analysis was performed using a one‐way ANOVA test. *P* < 0.05 (*), *P* < 0.01 (**), *P* < 0.001 (***), *P* < 0.0001 (****). Data are presented as mean ± SD. combo = bortezomib + pargyline.

When evaluating the scaffolds, both single agents and the combination therapy reduced the number of MM cells as indicated by a decrease in GFP positivity; however, the combination therapy did not further reduce the tumour load compared to bortezomib alone (Fig. 5A,B). In contrast, analysis of the cells in the supernatants revealed that the combination therapy significantly reduced the tumour load compared to either single agent (Fig. 5C). As proline plays a critical role in collagen synthesis, we also measured collagen deposition in the 3D model. Although a reduction in collagen staining was observed with the combination therapy, this decrease was not statistically significant (Fig. 5A,B). Next, the combination strategy was further evaluated in 2D coculture setups. First, we cocultured murine MS5 cells (BMSCs) and murine 5TGM1‐eGFP‐positive (MM) cells and simultaneously treated the cells with pargyline and bortezomib or the combination of both for 48 h. MM viability, as indicated by eGFP positivity, was significantly reduced and even close to 0% in the combination treatment group compared to control or monotherapies (Fig. 5D). Next, we wanted to further explore the effects of stromal PYCR1 inhibition by pretreating the MS5 cells with pargyline for 48 h before adding 5TGM1 cells and bortezomib. While the reduction in MM viability was smaller compared to the direct combination treatment, it remained significant compared to control and both single agents (Fig. 5E).

Finally, we have shown in our previous paper that pargyline enhances bortezomib‐mediated effects on tumour load *in vivo* in the 5TGM1 model [21]. To confirm the involvement of stromal PYCR inhibition on collagen deposition, we also measured collagen C‐terminal peptide, a marker for bone turnover, in the serum of the mice. However, no significant effect on collagen was observed with either compound (Fig. 5F). Since PYCR1 targeting in BMSCs reduces activin A release *in vitro*, we also measured activin A levels in the serum samples. Both pargyline treatment alone and in combination with bortezomib significantly reduced activin A levels compared to control, although this reduction was previously found to be insufficient to control tumour growth on its own (Fig. 5G).

## 4 Discussion

Numerous studies consider PYCR1 as a potential cancer therapy target due to its correlation with cancer progression [34, 35, 36, 37, 38, 39]. In our previous paper, we highlighted the role of PYCR1 and proline metabolism in MM [21]. High PYCR1 expression correlated with poor survival outcomes in MM, and its expression was even further upregulated after relapse. We showed that the inhibition of PYCR1 by either siRNA or the small molecule inhibitor pargyline reduced the proliferation and viability of the MM cells, accompanied by a decrease in protein synthesis. Importantly, PYCR1 interference enhanced bortezomib‐mediated cell death both *in vitro* and *in vivo* by upregulating the unfolded protein response pathway [21].

In this paper, we found that PYCR1 expression was not only high in MM cells, but also in BMSCs, which could be further enhanced by exposure to MM secretome. The mechanism by which MM cells upregulate PYCR1 remains unclear. Possibly, the MM CM upregulated FTH1, a known protector against ferroptosis, which is enhanced by the MM‐BMSC crosstalk and which has been shown to upregulate PYCR1 [40, 41]. Another possibility is that MM cells secreted factors such as FGF2, which enhance c‐MYC expression, which has also been correlated to PYCR1 levels [42, 43].

These data indicate that PYCR1 is not only involved in MM metabolism but also in MM‐BMSC crosstalk. It is known that BMSCs directly and indirectly (via their conditioned medium and secreted exosomes) stimulate MM cell growth and induce up to 1.5‐fold increase in drug resistance in recipient MM cells [44, 45, 46]. We therefore investigated whether we could reverse the protective effect of the BMSC secretome on MM cells via PYCR1 silencing and lower drug resistance. We cultured MM cells with the CM of BMSCs treated with siRNA against PYCR1. As a result, we observed a reduction in MM oxidative phosphorylation (OXPHOS), as indicated by a decrease in OCR values. A possible explanation for this is the fact that by blocking PYCR1, less proline is secreted by BMSCs. Since it is known that proline can be transformed back into glutamate, which can then enter the tricarboxylic acid cycle (TCA) cycle as α‐ketoglutarate [47], less available proline could result in less glutamate and α‐ketoglutarate, leading to lower function of the TCA cycle with lower concentrations of intermediates being formed as well as less availability of NADH and FADH_2_. As these two cofactors are necessary for the proper functioning of the electron transport chain, a reduction in proline availability could lead to lower OCR rates. Another possibility is that the Akt pathway is less activated in the MM cells, leading to less OXPHOS. Other groups have also shown that BMSCs support the progression of MM via metabolic processes. Matula et al. showed that stromal cells are able to transfer their mitochondria to MM cells via tubuli, thereby increasing the ATP production capacity and increasing drug resistance in the MM cells [48]. Additionally, researchers have shown that CAFs, a specific subtype of BMSCs, actively secrete multiple metabolites, such as pyruvate, lactate and amino acids into the environment, stimulating OXPHOS in the recipient cancer cells. This phenomenon is known as the ‘Reverse Warburg effect’ [49, 50, 51, 52, 53]. In relation to proline metabolism, Kay et al. have shown that breast cancer‐associated CAFs produce high levels of proline through PYCR1 to form a strong extracellular matrix, rich in collagen [22]. The inhibition of PYCR1 in CAFs by either shRNA or small molecule inhibitor pargyline reduced collagen production and tumour proliferation both *in vitro* and *in vivo*. Although we have also previously shown the tumour‐limiting effects of PYCR1 inhibition *in vivo*, we could not confirm a decrease in collagen deposition [21].

Inhibiting PYCR1 in BMSCs increased the sensitivity of the MM cells to bortezomib, a standard‐of‐care agent for MM treatment, likely by affecting OXPHOS levels. It has been shown that drug‐resistant cancer cells have a change in preferred metabolism, showing high levels of OXPHOS activity [54, 55]. Moreover, multiple studies have indicated that OXPHOS inhibition can make cancer cells more sensitive to anticancer agents. In lymphoma, Giuèze et al. demonstrated that venetoclax resistance was associated with increased OXPHOS and that inhibition of OXPHOS by antimycin or oligomycin resensitised cells to venetoclax [56]. Also, cells from AML patients with IDH mutations showed increased OXPHOS, whereby their inhibition enhanced their response to IDH inhibitors [57]. Finally, we have also previously identified that metformin, a complex I inhibitor of OXPHOS, sensitises MM cells to the glycolysis inhibitor syrosingopine in MM [58]. Combining an OXPHOS inhibitor with other anticancer drugs was also successful in melanoma [59] and ovarian cancer [60].

Unexpectedly, we demonstrate here for the first time that PYCR1 inhibition in BMSCs also decreases the release of activin A. Activin A is a member of the TGF‐β superfamily, and involved in embryogenesis, gonadal hormone signalling and more importantly in MM, bone remodelling [11, 61, 62]. Out of all factors which are part of the TGF‐β superfamily, activin A has one of the highest concentrations in bone, and acts as a paracrine hormone to stimulate the activity of osteoclasts [63, 64]. The role of activin A has previously been studied in MM. Vallet et al. found increased levels of activin A in MM patients suffering from osteolytic lesions, while it also inhibited the differentiation of osteoblasts, further stimulating osteolytic disease [11]. Inhibition of activin A improved MM bone disease and reduced tumour growth *in vivo*. Other research groups have also shown the role of activin A in supporting cancer growth and migration. However, in another study in MM, low concentrations of activin A reduced viability and increased apoptotic cell death of the uncommon murine MM cell line NS‐1 [65]. This is remarkable as we observed the opposite effect on human OPM2 cells. Although we used higher concentrations, comparable to Vallet et al., it has been shown that activin A is able to exert a dual role, as it promotes tumorigenesis and proliferation in some cancers, but can induce apoptosis in others. For example, activin A initiated apoptosis or blocked proliferation in breast cancer [66], prostate cancer [67] and diffuse large B cell lymphoma [68] among others, while it stimulated tumour growth in malignant pleural mesothelioma [69] and, similar to our observations, in lung fibroblasts of lung cancer patients [70, 71].

Lastly, we aimed to demonstrate the clinical potential of targeting PYCR1 in MM by using the PYCR1 inhibitor pargyline in relevant model systems. To include the stromal compartment *in vitro*, we used a 3D coculture system whereby BMSCs were seeded on a 3D scaffold, after which MM cells were added. We only observed enhanced tumour killing, compared to bortezomib alone, in the supernatants, which further indicates that pargyline is mainly affecting the secretome of the BMSCs. Since clinical implementation of PYCR1 inhibitors might be impeded by off‐target toxic effects, we suggest using targeted delivery of small molecule inhibitors, specifically to the BM environment. Menachem et al. bypassed off‐target toxic effects of bortezomib by loading the compound into liposomes, a strategy shown to achieve better therapeutic efficacy compared to free bortezomib, even in bortezomib‐resistant MM clones [72]. A similar strategy could be used for pargyline.

Since quantifying the adherent cells in the 3D scaffold proved difficult, we continued with different 2D coculture setups of BMSCs and MM cells. Herein, the combination therapy significantly reduced viability compared to both single agents. This is in line with our previous research that showed the direct anti‐MM effects of pargyline in a combination setting with bortezomib, however without BMSCs included [21]. As BMSCs are known to induce drug resistance, it is encouraging to still observe strong antitumour effects in coculture settings, which were even more pronounced upon stromal PYCR1 inhibition. When further examining pargyline effects *in vivo*, we confirmed a significant decrease in serum activin A levels upon pargyline treatment, but no breakdown of collagen.

## 5 Conclusions

In conclusion, we demonstrate here for the first time that PYCR1 inhibition in stromal cells inhibits OXPHOS in MM cells and sensitises MM cells to the proteasome inhibitor bortezomib and reduces activin A release. As we have also previously shown the antitumour effects of pargyline in addition to bortezomib *in vivo*, we propose PYCR1 as a potential target not only in the MM cells but also in their tumour microenvironment.

## Conflict of interest

We hereby guarantee that the work performed by Dr. Judith Lind was carried out as described. I, as corresponding author, am accountable for the accuracy and integrity of the work performed. I confirm that Dr. Lind has no conflict of interest.

## Author contributions

EM and IO: conceptualization. IO, EM, LvdB; OA, JL, SV, GA and KP: methodology and investigation. EM, KV and KP: resources. IO, LvdB, EM, OA and JL: formal analysis. IO, LvdB and EM: writing‐original draft preparation. EM, IO, LvdB, OA, JL, SV, AvdV, GA, AM, KM, KDV, EDB, KV and KP: Writing‐Review and Editing. EM and KP: supervision. The author(s) read and approved the final manuscript.

## Supporting information


**Fig. S1.** PYCR1 knockdown by siRNA in BMSCs does not affect its viability.
**Fig. S2.** PYCR1 inhibition in HT1080 does not sensitise MM cells to bortezomib treatment in co‐culture, showing specific MM‐BMSC effects.
**Fig. S3.** PYCR1 inhibition in stromal cells combined with bortezomib lowers OXPHOS in MM.

## Data Availability

Source data are provided with this paper. All other data supporting the findings of the study are available from the corresponding authors upon request.
