# Supplementary material for: PYCR1 inhibition in bone marrow stromal cells enhances bortezomib sensitivity in multiple myeloma cells by altering their metabolism
Source: Mol Oncol. 2025 Sep 11;20(2):447–63. doi: 10.1002/1878-0261.70120 (PMC12936435; doi:10.1002/1878-0261.70120)
Supplement: Supplementary file 1 — Fig. S1. PYCR1 knockdown by siRNA in BMSCs does not affect its viability. Fig. S2. PYCR1 inhibition in HT1080 does not sensitise MM cells to bortezomib treatment in co‐culture, showing specific MM‐BMSC effects. Fig. S3. PYCR1 inhibition in stromal cells combined with bortezomib lowers OXPHOS in MM. [file MOL2-20-447-s001.docx]

**Supplementary figures**


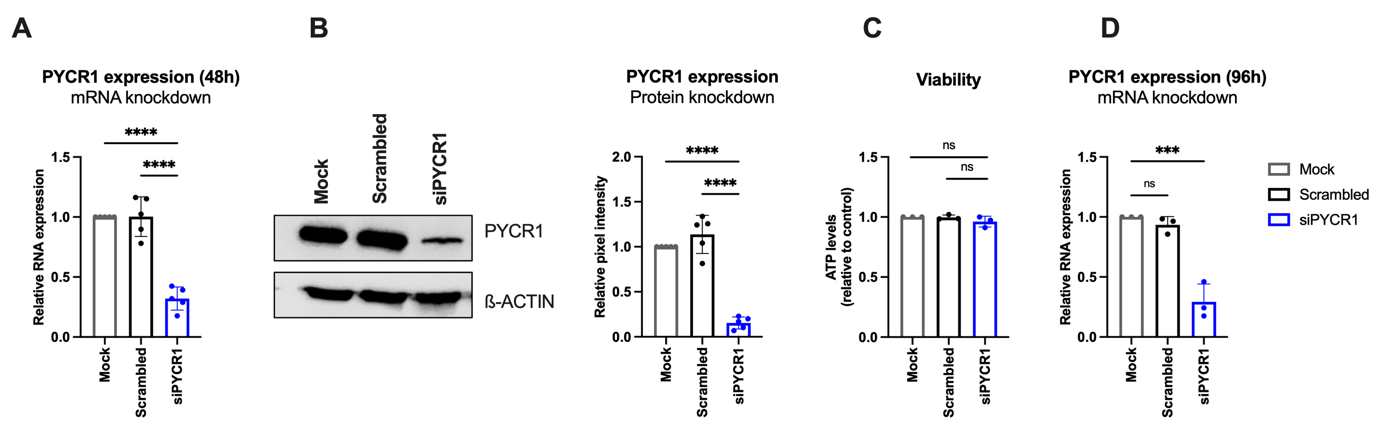


***Suppl Figure 1: PYCR1 knockdown by siRNA in BMSCs does not affect its viability. (A)*** *HS5 cells were treated with siPYCR1 or control (mock, Scrambled) and cultured for 48h. PYCR1 knockdown was verified by RT-qPCR on RNA level (n=5)* ***(B)*** *PYCR1 protein knockdown was verified by western blot (n=5)* ***(C)*** *Viability of HS5 cells was measured by CellTiterGlo assay (n=3).* ***(D)*** *PYCR1 mRNA knockdown was verified by qRT-PCR after 96h (n=3). Statistical significance was measured by a one-way ANOVA.*


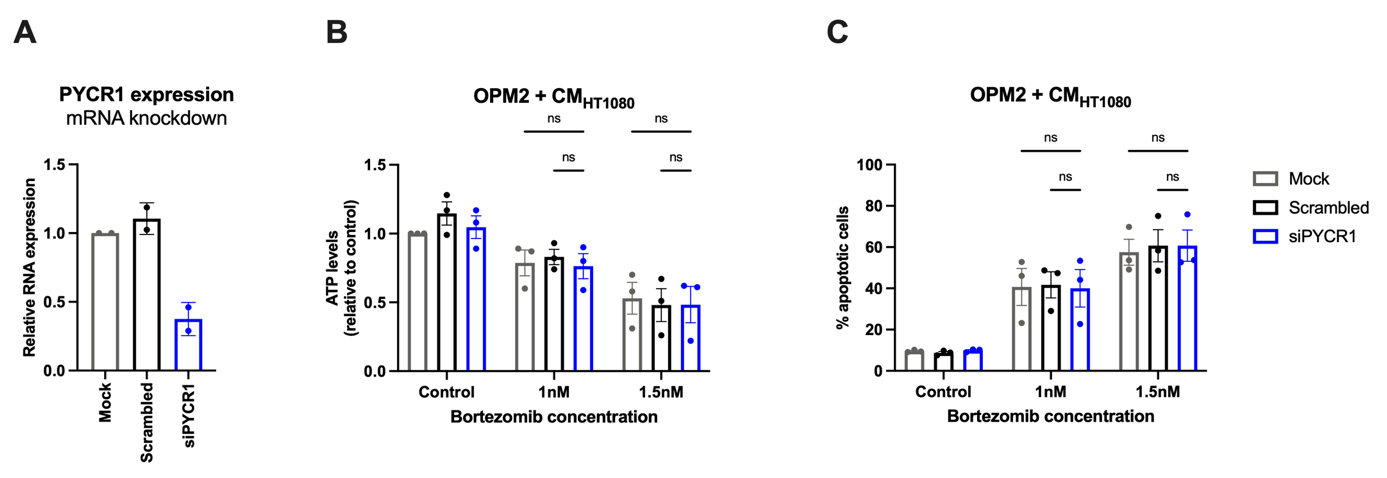


***Suppl Figure 2:*** ***PYCR1 inhibition in HT1080 does not sensitise MM cells to bortezomib treatment in co-culture, showing specific MM-BMSC effects. A)*** *HT1080 were treated with siRNA against PYCR1 (siPYCR1) or controls (Mock; scrambled) and their CM was collected after 48h and used as treatment for OPM2 cells. PYCR1 mRNA knockdown was measured using qRT-PCR. (n=2)* ***B)*** *Viability was measured in OPM2 cells cultured with siPYCR1-CM or control using CellTiterGlo assay. (n=3)* ***C)*** *Apoptosis was measured in OPM2 cells cultured with siPYCR1-CM or controls using flow cytometry (n=3). Statistical significance was measured by a repeated-measures one-way ANOVA test.*


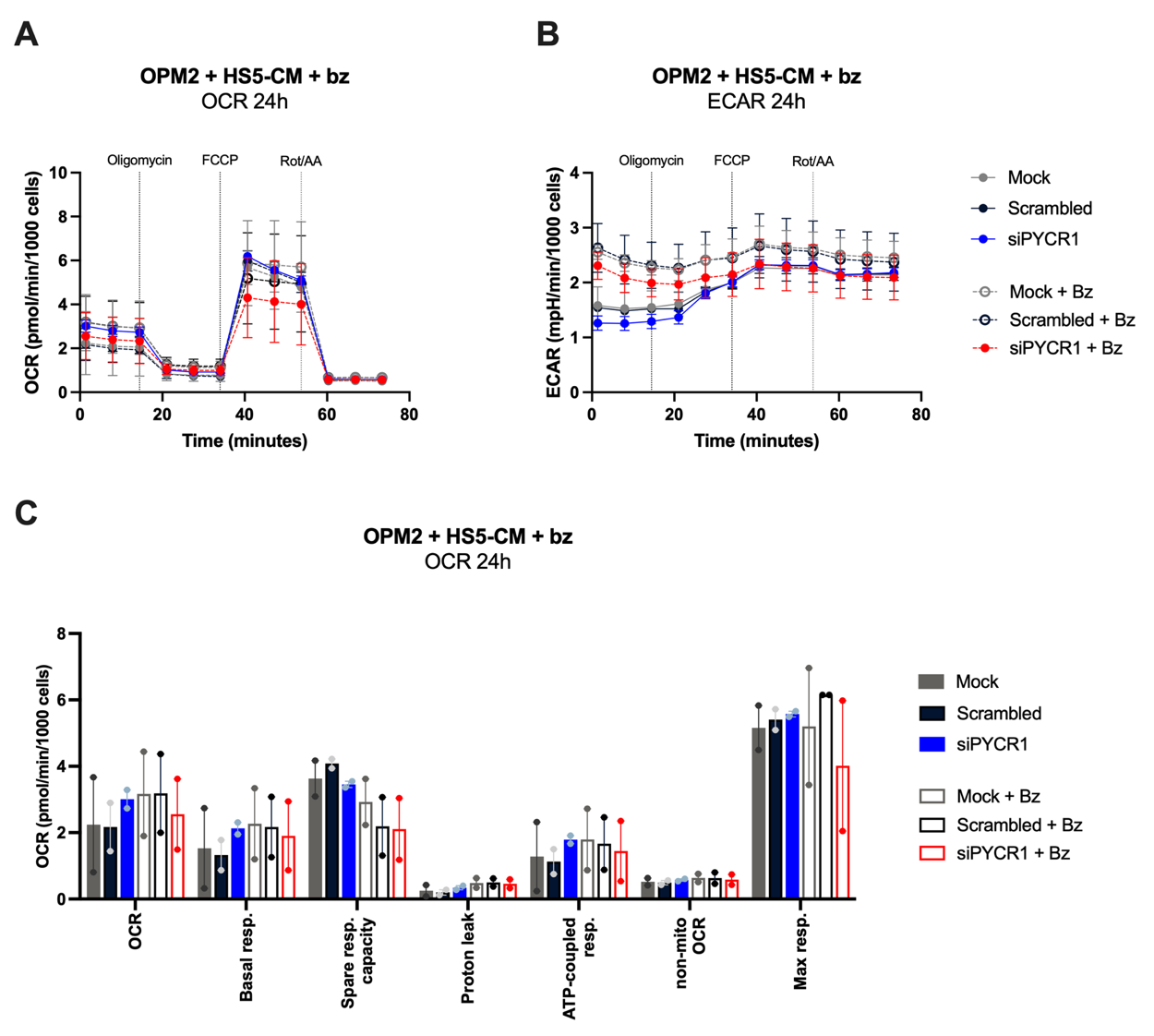


***Suppl Figure 3: PYCR1 inhibition in stromal cells combined with bortezomib lowers OXPHOS in MM. (A-B)*** *OCR and ECAR rates measured in real-time after OPM2 cells were treated for 24h with CM isolated from PYCR1-inhibited HS5 cells (siPYCR1) or control (mock, scrambled) and bortezomib (n = 2).* ***(C)*** *Overview of multiple OXPHOS-related parameters (n = 2). CM = conditioned medium, OCR = oxygen consumption rate, ECAR = extracellular acidification rate, Rot/AA = rotenone/antimycin A, FCCP = phenylhydrazone, resp. = respiration, bz = bortezomib.*
